# BCDP: Budget Constrained and Delay-Bounded Placement for Hybrid Roadside Units in Vehicular *Ad Hoc* Networks

**DOI:** 10.3390/s141222564

**Published:** 2014-11-27

**Authors:** Peng Li, Chuanhe Huang, Qin Liu

**Affiliations:** 1 Computer School, Wuhan University, Luo-Jia-Shan Road 16, Wuhan 430072, China; E-Mails: lip@whu.edu.cn (P.L.); qinliu@whu.edu.cn (Q.L.); 2 College of Computer Science and Technology, Wuhan University of Science and Technology, Heping Road 947, Wuhan 430081, China

**Keywords:** roadside unit, facility placement, delay bound, vehicular sensor networks

## Abstract

In vehicular *ad hoc* networks, roadside units (RSUs) placement has been proposed to improve the the overall network performance in many ITS applications. This paper addresses the budget constrained and delay-bounded placement problem (BCDP) for roadside units in vehicular *ad hoc* networks. There are two types of RSUs: cable connected RSU (c-RSU) and wireless RSU (w-RSU). c-RSUs are interconnected through wired lines, and they form the backbone of VANETs, while w-RSUs connect to other RSUs through wireless communication and serve as an economical extension of the coverage of c-RSUs. The delay-bounded coverage range and deployment cost of these two cases are totally different. We are given a budget constraint and a delay bound, the problem is how to find the optimal candidate sites with the maximal delay-bounded coverage to place RSUs such that a message from any c-RSU in the region can be disseminated to the more vehicles within the given budget constraint and delay bound. We first prove that the BCDP problem is NP-hard. Then we propose several algorithms to solve the BCDP problem. Simulation results show the heuristic algorithms can significantly improve the coverage range and reduce the total deployment cost, compared with other heuristic methods.

## 1. Introduction

Vehicular *ad hoc* networks (VANETs) are a type of networks in which vehicles and roadside units(RSUs) are the communicating nodes, providing each other with information, such as safety warnings and traffic information [1]. A vehicle can communicate with other vehicles either directly if they are within each other's transmission range or through the RSUs otherwise. These nodes are wireless nodes with store-and-forward capabilities placed along vehicles' routes that exchange data with vehicles that pass through the node. Therefore, they increase the opportunities of connectivity in network and improve the overall network performance. RSUs act similar to a wireless LAN access point and can provide communications with infrastructure if budget is enough. Due to the stationary characteristic, people could place infrastructure-less RSUs in order to enhance coverage probability if budget is not enough. As RSUs are wireless nodes with store-and-forward capabilities placed along vehicles' routes that exchange data with vehicles that pass through the node, there are some approaches which exploit RSUs help routing [2,3], geocasting [4] and data delivery [5,6]. Meanwhile, the proper placement of c-RSUs and w-RSUs is crucial for the new generation ITS applications, such as content downloading [7–9], content distribution [10–13], and distributed road video surveillance [14,15]. In a word, the overall performance can be improved by placing additional RSUs in the vehicular networks.

Several RSUs or AP placement algorithms have been proposed [16–19]. Most of these approaches consider the coverage and/or the connectivity of network. There are many applications that rely on delay-bounded information dissemination in vehicular networks such as unmanned automobile and safe driving [20–22]. However, few research considers the delay-bounded placement problem. In this paper, we study the budget constrained and delay-bounded placement problem. Given a budget constraint and a delay bound, if we place too little RSUs, the delay bound cannot be met; but if we place too many RSUs, the budget constraint cannot be met. Meanwhile, each RSU has two cases for backbone access: cable connected RSU (c-RSU) and wireless RSU (w-RSU). c-RSUs are interconnected through wired lines, and they form the backbone of VANETs. They also usually have a larger communication range due to the availability of power source and more powerful devices. Despite the benefit of fast information dissemination, c-RSUs are often associated with high placement cost. On the other hand, w-RSUs connect to other RSUs through wireless communication and typically have a smaller transmission range. They serve as an economical extension of the coverage of c-RSUs.

The problem of our concern is as follows. We are given a set of candidate sites in a region, each of which can be installed a c-RSU or a w-RSU (or none). The candidate site is usually located at the intersection of roads or along the road, such as a lamp post or a tree. Given a budget constraint and a specified delay bound, the problem is how to place the c-RSUs and w-RSUs with the maximum delay-bounded coverage, such that a message sent from the c-RSUs can be disseminated to more vehicles in the region within the delay bound.

There are several challenges for solving the budget constrained and delay-bounded problem in a VANET. First, we consider the placement of hybrid RSUs, that for each candidate site we need to decide what type of RSUs to choose, which is more complicated than the placement of the same type of RSUs; Second, we study the placement of RSUs for the delay bounded information dissemination, which is required by many real-time applications in vehicular networks. The main contributions of the paper are as follows:

- We propose a delay-bounded coverage model for RSUs placement, in which each candidate site has an attached delay-bounded coverage by installing a RSU. This model inherently provides a good balance between delay and coverage. Base on this model, we formulate the BCDP problem to a budgeted maximum coverage problem and prove that this problem is NP-hard.
- We develop two heuristic algorithms under delay-bounded coverage model. In this model, we greedily find the optimal candidate site with a maximum delay bounded coverage gain and utility until no further installing can be made due to the budget constraint.
- We conduct an extensive simulation study over different data to evaluate the performances of the proposed methods. Simulation results show our heuristic algorithms can significantly improve the coverage range and reduce the total installation cost, compared with other heuristic methods.

The rest of this paper is organized as follows. Previous studies are summarized in Section 2. We present the system model and assumption in Section 3. The detailed description of our delay-bounded coverage model and our greedy algorithms are in Sections 4 and 5, respectively. In Section 6, we evaluate our proposed approaches by comparing them with different simulation environment. Our conclusion is in Section 7.

## 2. Related Work

### 2.1. RSUs-Aided Data Delivery in VANET

Recently, the research have attracted a lot of attention on the data delivery and message dissemination in vehicular *ad hoc* networks [23–25]. As these studies consider inter-vehicle data delivery in a vehicular network in which there is no infrastructure support, and some other studies consider a vehicular network with an infrastructure [3–7,26,27]. Wu *et al.* [3] investigated the optimal infrastructure-assisted routing for inter-vehicle data delivery. They considered packet forwarding and buffer allocation problem and designed a global algorithm to solve the optimization problem. Mershad *et al.* [4] exploited the infrastructure of roadside units (RSUs) to efficiently and reliably delivery message to far vehicles. Ding and Xiao [5] proposed that vehicles delivered data with the help of RSUs. Jeong *et al.* [6] exploited the RSUs and relay nodes to efficient data delivery in vehicle networks. In TBD [26] and TSF [27], they considered data delivery from vehicles to fixed APs or from APs to vehicles. Meanwhile, Malandrino *et al.* [7] investigated that optimal AP deployment over the street layout could help content downloading. In [8,9], the authors proposed several cooperative downloading protocols, maximized the data rate of data flow or amount of data packets downloaded from the RSU. Li *et al.* [10] studied the problem of multiple content dissemination under realistic RSU-aided opportunistic networks and proposed an efficient heuristic algorithm to solve this problem. Zhang *et al.* [11] exploited infrastructure APs to collaboratively distribute contents to moving vehicles and attempted to maximize the vehicular download performance obtained from the infrastructure of APs. Wang *et al.* [12] proposed a coalition formation game model to solve the content distribution problem, which provided a new method for solve the content distribution problem. Also, there are some published work related to delay-bounded routing problem [28–30]. Skordylis *et al.* [28] addressed the delay bounded routing through the proposal of two algorithms, which forwarded packets to APs satisfy the user-defined delay bound rather than the lowest delivery delay. In [29], the authors investigated the broadcast storm problem and proposed effective warning delivery algorithm. The work in [30] proposed a Delay-Aware Data Delivery (DADD) scheme to achieve delay minimal bundle delivery from source to destination. All above work can be useful for our delay bounded placement problem.

### 2.2. Node Placement in Wireless Networks

There are some published work about the node and AP placement problem in wireless sensor networks [31–38]. These previous researches could be classified into the following categories. Based on the routing structure, the researches classified into single-tiered and two-tiered. In the single-tired category, each sensor node not only forwards the packet to the relay nodes, but also forwards the packet to the other sensor nodes. In the two-tired category, each sensor node only forwards its sensor information to the relay nodes, but dose not forward the packet to the other sensor nodes. Based on the network connectivity, the researches classified into 1-connectivity or k-connectivity. For 1-connectivity relay node placement, the placement of relay nodes ensures the 1-connectivity between the sensor nodes. For k-connectivity relay node placement, the placement of relay nodes ensures the k-connectivity between the sensor nodes and relay nodes. Liu *et al.* [32] propose several approximation algorithms to solve the minimum relay-node placement problem in two tiered wireless sensor networks for 1-connectivity and 2-connectivity. Lloyd and Xue [33] also studied the single-tiered relay node placement problem and two-tiered relay node placement problem, and presented several polynomial time algorithms for the problems. Zhang *et al.* [34] investigated four relay node placement problems for k-connectivity and proposed several polynomial time algorithms to solve the problems.

Previous work addressed the problem of relay nodes placement in homogeneous wireless sensor networks. Han *et al.* [35] addressed the problem of relay nodes placement in heterogeneous wireless sensor networks. In [36,37], the authors studied the constrained relay node placement problem, that the relay nodes can be placed in some given candidate sites. Liu *et al.* [38] addressed the problem in which the network cost is minimized while the resulting lifetime is at least equal to a given value, and they placed the minimum number of sensors such that all the given targets can be monitored. Also, there are some published work about the node problem in wireless networks [39–41]. Zhang *et al.* [40] studied the optimal placement of APs such that the total cost of all APs is minimized, subject to the constraint that the traffic demand for each client can be fulfilled. Zhou *et al.* [41] investigated the minimal number of APs placement problem, so that both fault tolerance and QoS constraints can be satisfied. All above node placement studies are most related to our problem.

### 2.3. RSUs Placement in VANETs

Meanwhile, there are some published work related to the problem of RSUs or stationary relay nodes placement in vehicular *ad hoc* networks [16–19,42–45]. In [16], authors addressed the problem of optimally placing gateways in vehicular networks to minimize the average number of hops from access points (APs) to gateways. This work was extended in [17], where the authors showed that the problem of optimal relay node placement is a NP-hard problem and proposes an integer linear programming (ILP) formulation for this problem. In [18], the authors presented an efficient deployment method that maximizes the worst case contact opportunity under a budget constraint. The work of [19] studied the problem of optimal wireless relay placement (RPP) for sensory data collection from the vehicles. The authors in [42] continued the previous work and proved that it is NP-hard to compute the minimum number of base stations for achieving the full coverage. In [43], the authors chosen the interconnection gap as a metric for optimization to solve the APs placement problem. The work in [44,45] are most related to our problem. The work of [44] studied two AP deployment problems that aim at maximizing the continuous user coverage and minimizing the AP deployment cost, and proved both problems are NP-complete. Reference [45] considered three cases placement problem, such as cellular BSs, wireless mesh backbones (WMBs), and roadside access points (RAPs). However, delay-bounded placement is not considered in prior research.

The difference between the above work with ours is that, we consider the delay-bounded placement problem, rather than minimize the number of RSUs in vehicular networks. Meanwhile, we consider the optimal placement scheme for the both infrastructure-based and infrastructure-less RSUs. To the best of our knowledge, our work is the first to study this problem in the literature.

## 3. System Model and Assumption

A VANET system consists of a set of vehicles equipped with on-board units (OBUs) and a set of roadside units (RSUs) that are installed at the road side. The system model and assumption are as follows:

- We assume all c-RSUs have the same communication range, and the communication range is larger than the w-RSUs. The warning information, such as traffic accident or vehicle jam, disseminates from the traffic control center(TCC) to all c-RSUs firstly. TCC is a centralized controller that maintains the city's traffic. Then, the message spreads to the w-RSUs through wireless link. In the end, the message floods from c-RSUs and w-RSUs to all the vehicles.
- In the case of c-RSUs, we assume the information delay from TCC to all c-RSUs doesn't take any time (there is no delay). The assumption is acceptable as all c-RSUs have the wired network connectivity to the TCC, and the information delay almost can be ignored.
- In the case of w-RSUs, we further assume that a w-RSU only receives the message from a nearby c-RSU or w-RSU, but never gets the message from a passing vehicle. Once receiving a message, a w-RSU would broadcast the message immediately. Thus, a w-RSU is connected to other RSUs to serve as an effective extension to the backbone formed by c-RSUs.
- For information dissemination, the vehicle receives a message from an RSU when it passes the RSU, and retrieves the “fresh” messages stored in the RSU and carry it forward for further dissemination. A moving vehicle on the road can also pass its messages to other vehicles via the OBUs equipped them. In this case, we call it the carry-and-forward delay. Due to carry-and-forward delay several orders of magnitude longer than the communication delay, we assume that the communication delay also approximately equals 0. The assumption is acceptable as the unit of carry-and-forward delay is a second and the unit of communication delay is a millisecond.

We model a road network as a undirected weighted road graph *G* = (*V*, *E*), while the vertex set *V* (*G*) = {*v*_1_, *v*_1_, …, *v_m_*} consists of all candidate sites, and the number of candidate sites is *m*; the edge set *E*(*G*) = {*e*_1_, *e*_1_, …, *e_n_*} consists of the entire road segments, and the number of road segments is *n*. Each edge is associated with a delay weight *d_ij_*, which is the expected delay from *v_i_* to its adjacent vertex *v_j_*. That means *v_i_* is connected to *v_j_* an edge in a road graph, and we call *v_i_* and *v_j_* are neighbors. According to the [5,25], we have:

(1) $$E[d_{ij}]=(1-e^{-R\cdot \rho_{ij}})\cdot \frac{l_{ij}\cdot c}{R}+e^{-R\cdot \rho_{ij}}\cdot \frac{l_{ij}}{{\bar{s}}_{ij}}$$

where *R* is the wireless transmission range, *ρ_ij_* is the vehicle density between road *e*(*v_i_*, *v_j_*), *c* is the average one-hop packet transmission delay, *l_ij_* is the Euclidean distance of road *e*(*v_i_*, *v_j_*), and *s̄* is the average vehicle speed on road *e*(*v_i_*, *v_j_*).

**Figure 1.**
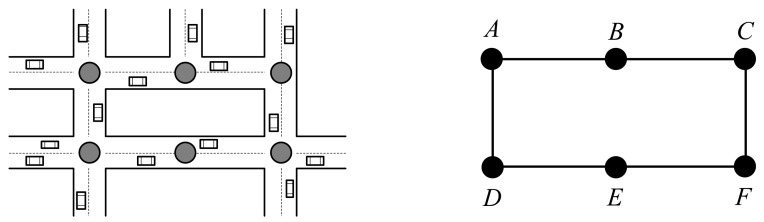
An example of weighted road graph.

As shown in Figure 1, the vertex is either on the intersection or along the road side when the road is long enough, such as the vertex *v_B_* and *v_E_*.

## 4. Delay-Bounded Coverage and Problem Formulation

In this section, we present the delay-bounded coverage model based on the road graph. In the model, we discuss in detail how to calculate the delay-bounded coverage by installing RSUs. Then, we formally formulate the BCDP problem based on the delay-bounded coverage model.

### 4.1. Information Dissemination Delay

As the benefit of candidate site *i* associated with the delay-bounded coverage, we calculate the information propagation delay first. Let *D_ij_* denote the expected information dissemination delay for broadcasting a message to all vehicles on the path between any candidate sites *i* and *j*. When *v_i_* and *v_j_* are neighbors, *D_ij_* equals to the delay *d_ij_*. If *i* and *j* do not have a road directly connecting them, *D_ij_* is the shortest path between *v_i_* and *v_j_*. Notice that *D_ij_* is calculated by assuming there is no RSU installed in the middle along the route between *i* and *j*. Let *N*(*i*) denote all the neighbors of node *i*, we calculate *D_ij_* as follows:

(2) $$D_{ij}=\{\begin{array}{ll} 0, & \text{if}\;j=i\\ d_{ij}, & \text{else if}\;j\in N(i)\\ \min_{k\in N(i)}\{D_{ik}+D_{kj}\}, & \text{otherwise} \end{array}$$

Next, we introduce the calculation of delay-bounded coverage for installing a i-RSU or a w-RSU, respectively. Then, we calculate the placement cost.

### 4.2. Delay-Bounded Coverage

Now we calculate the delay-bounded coverage for installing an RSU. We give some definitions as follows.

#### Definition 1

*Communication Set of vertex v_i_, denoted by* 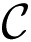
(*i*), *is a set of vertex that if we install an RSU in site v_i_, any vertex in* 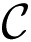
(*i*) *is within the communication range of the vertex v*_i_.

If we install an RSU in site *v_i_*, and the candidate site *v_j_* is within the communication range of *v_i_*, then we have *v_j_* ∈ 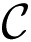
(*i*). We denote 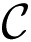
*_c_*(*i*) and 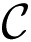
*_w_*(*i*) as the communication set of installing a c-RSU and a w-RSU at site *v_i_*, respectively.

#### Definition 2

*Delay-bounded Coverage of a vertex v_i_, denoted by* 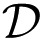
(*i*), *is a set of edge that if we install a c-RSU in site v_i_, a vehicle at any edge e*(*j, k*) ∈ 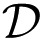
*_i_ would receive the message within the delay bound T*.

According to Definition 2, we have

(3) $$D(i)=\{e(j,k)|D_{ij}+d_{jk}\leq T\vee D_{ik}+d_{kj}\leq T\forall v_{j},v_{k}\in V(G),e(j,k)\in E(G)\}$$

We denote 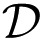
*_c_*(*i*) and 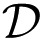
*_w_*(*i*) as the delay-bounded coverage of installing a c-RSU and a w-RSU at site *v_i_*, respectively. We denote 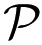
*_c_* and 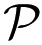
*_w_* as the c-RSUs placement set and w-RSUs placement set, respectively. We denote 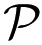 = 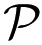
*_c_* ∪ 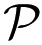
*_w_* as the RSUs placement set. Then we give the following definition.

#### Definition 3

*Communication Set of placement set* 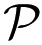, *denoted by* 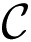
_
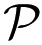
_, *is a set of placement set* 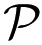 *that the expected end – end propagation delay from one vertex to any other vertex which are both in set* 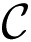
_
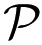
_ *is zero*.

According to the Definition 3, we calculate the communication set 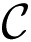
_
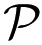
_ of placement set 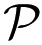 iteratively. We denote 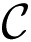
^(^*^k^*^)^ as the communication set value of k-th iteration. Initially, the placement set 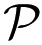 = ∅ and the communication set 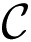 = ∅. When we place an RSU at site *i*, the placement set 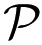 = {*v_i_*}. The communication set 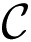
_
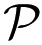
_ equals to the communication set of *v_i_*. That is $C_{P}^{\left(1\right)}=C(i)$. Next, we place another i-RSU at site *j*, then the placement set 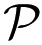 = {*v_i_, v_j_*}. Due to that we don't consider the delay between wired line, site *i* receives the information from site *j* in no time. Therefore, the communication set 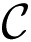
_
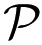
_ equals to the union set of transmission range set of *v_j_*, that is $C_{P\cup \{v_{j}\}}^{\left(2\right)}=C_{P}^{\left(1\right)}\cup C(j)$. Therefore, we have

(4) $$C_{P\cup \{v_{i}\}}^{\left(k\right)}=\{\begin{array}{ll} C(i), & k=1\\ C_{P}^{\left(k-1\right)}\cup C(i) & k>1 \end{array}$$

#### Definition 4

*Delay-bounded Coverage of placement set* 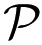, *denoted by* 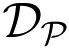, *is a edge set of placement set* 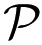 *that a vehicle at any edge e*(*j, k*) ∈ 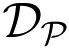 *would receive the message within the delay bound T*.

According to the Definition 4, we have:

(5) $$D_{P\cup \{v_{i}\}}^{\left(k\right)}=\{\begin{array}{ll} D(i), & k=1\\ D_{P}^{\left(k-1\right)}\cup D(i) & k>1 \end{array}$$

**Figure 2.**
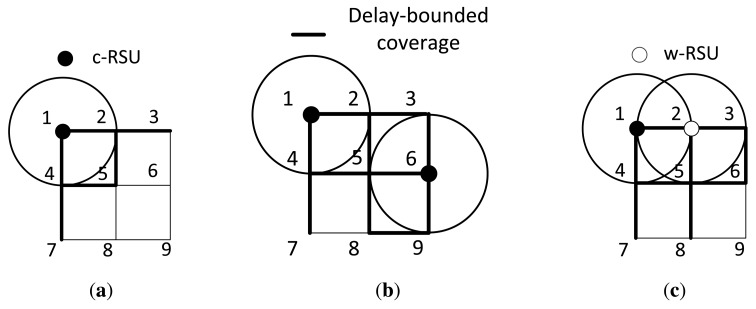
An example of transmission range set and delay-bounded coverage. (**a**) The first iteration; (**b**) The second iteration for installing a cable connected roadside unit (c-RSU); (**c**) The second iteration for installing a wireless RSU (w-RSU).

Where 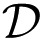
^(^*^k^*^)^ as the delay-bounded coverage of the k-th iteration. Figure 2 presents an illustrative example of the communication set and the delay-bounded coverage. The system has nine candidate nodes *v*_1_ to *v*_9_, and we model the system as a undirected weighted road graph *G*. In graph *G*, each edge has the information dissemination delay of weight 1 unit and each RSU has also 1 unit communication range. Then, we could use Equation (2) to calculate information propagation delay from source site to destination site. In the first iteration, as shown in Figure 2a, we place a c-RSU in the first iteration at the vertex *v*_1_, and we obtain the placement set 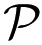 = 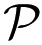
*_c_* = {*v*_1_}. Since vertex *v*_1_, *v*_2_ and *v*_4_ are within the communication range of *v*_1_, the communication set $C_{P}^{\left(1\right)}=C_{c}(1)=\{v_{1},v_{2},v_{4}\}$. For simplicity, we assume the delay bound *T* = 1 unit. Then, we could derive the delay-bounded coverage $D_{P}^{\left(1\right)}=D_{c}(1)=\{e(1,2),e(1,4),e(2,3),e(2,5),e(4,5),e(4,7)\}$. In the next iteration, we assume to place the next c-RSU in the vertex *v*_6_ as shown in Figure 2b, and there is no delay between *v*_1_ and *v*_6_ due to cable connected between them. As the placement set is 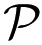 = {*v*_1_, *v*_6_} and the communication set of *v*_6_ is 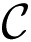
*_c_*(6) = {*v*_3_, *v*_5_, *v*_6_, *v*_9_}, we iteratively derive the communication set $C_{P}^{\left(2\right)}=C_{P}^{\left(1\right)}\cup C_{c}(6)=\{v_{1},v_{2},v_{3},v_{4},v_{5},v_{6},v_{9}\}$. We also calculate the delay-bounded coverage of *v*_6_ is 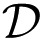
*_c_*(6) = {*e*(2, 3), *e*(2, 5), *e*(3, 6), *e*(5, 6), *e*(5, 8), *e*(6, 9), *e*(8, 9)}, and the delay-bounded coverage of this iteration is $D_{P}^{\left(2\right)}=D_{P}^{\left(1\right)}\cup D_{c}(6)=\left\{e(1,2),e(1,4),e(2,3),e(2,5),e(3,6),e(4,5),e(4,7),e(5,6),e(5,8),e(6,9),e(8,9)\right\}$. Since we all install the c-RSU in this two iteration, the placement set 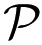 = 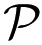
*_c_* = {*v*_1_, *v*_6_}. As shown in Figure 2c, if we install a w-RSU in the second iteration, we have 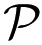
*_w_* = {*v*_2_} and 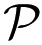
*_c_* = {*v*_1_}, and the black thick lines are the delay-bounded coverage road segments in this iteration.

### 4.3. Problem Formulation

Our problem can be viewed as a Budget Constrained and Delay-bounded RSUs Placement problem (BCDP for short) over road graphs, that is: *Given a budget constraint B, a delay bound T, a set of candidate sites, our goal is to find a candidate sites optimal set such that provides maximum delay-bounded coverage in a delay bound T with the given budget constraint B*.

We denote *c_i_* and $c_{i}^{\prime }$ as the placement cost for site *i* to be installed a c-RSU and a w-RSU respectively Obviously, the placement cost of installing c-RSUs or w-RSUs is totally different. It is known that each c-RSU installation with power and wired network connectivity can cost as high as US$5000 [46]. Let *X* = {*x*_1_, *x*_2_, …,*x_n_*} denote whether site *i* will be installed a c-RSU or not. If site *i* is selected to install a c-RSU then *x_i_* = 1, otherwise *x_i_* = 0. In a similar way, we denote *Y* = {*y*_1_, *y*_2_,…,*y_n_*} whether site *i* will be installed a w-RSU or not.

Therefore, our objective is to maximize the delay-bounded coverage of placement RSUs in the region, that is:

(6) $$\max|D_{P}|$$

subject to:

(7) $$\sum_{i\in V}(x_{i}\cdot c_{i}+y_{i}\cdot c_{i}^{\prime })\leq B,\forall i,j\in V$$

(8) $$x_{i},y_{i}\in \{0,1\},\forall i\in V$$

(9) $$x_{i}+y_{i}\leq 1,\forall i\in V$$

Constraint (7) states that the sum of placement cost should not exceed the budget constraint *B*. Constraint (8) defines the decision variables *x, y* as binary. Constraint (9) indicates that there is at most one c-RSU or w-RSU to install at each candidate site.

The BCDP problem is difficult to solve, as shown in the following theorem.

#### Theorem 1

*The BCDP problem is NP-hard*.

##### Proof

To provide the evidence of NP-hardness, we provide a polynomial reduction from a budgeted maximum coverage problem [47] to the BCDP problem.

A budgeted maximum coverage problem (*U; S; c*): Let *U* = {*e*_1_, *e*_2_, …*e_n_*} denote a set of *n* elements, *S* consists of finite sets *S*_1_, *S*_2_, …, *S_k_* ⊆ *U*, and a cost function *c* : *S* → *Q*^+^. The objective is to find a subset *S*′ ⊆ *S*, such that the total cost of elements in *S*′ does not exceed a given budget *B*, and the total weight of elements covered by *S*′ is maximized.

We next show the budgeted maximum coverage problem can be reduced to our problem. Let each element *e_i_* ∈ *U* correspond to the candidate site *v_i_* ∈ *V, S_k_* denotes the delay-bounded coverage of *v_i_* and the cost function *c* denotes the corresponding cost function of placing an RSU in the candidate site *v_i_*. Therefore the objective of our problem is to find an placement set of candidate site to place RSUs with maximum delay-bounded coverage of placement set and the total cost of elements in *S*′ does not exceed a given budget, which is just to find a subset of *S* with maximum total weight of elements covered by *S*′ in budgeted maximum coverage problem. After that, we reduce the BCDP problem to a budgeted maximum coverage problem in the polynomial time.

## 5. Our Solution

In this section, we proposed two greedy algorithms to solve the BCDP problem. One is to find the maximum delay-bounded coverage gain, and the other is to find the maximum delay-bounded coverage utility. Then, we analysis the approximation ratio and time complexity of proposed utility greedy algorithm.

### 5.1. Gain Greedy Algorithm

As proven in Theorem 1, we reduce the BCDP problem to the budgeted maximum coverage and prove that the problem is NP-hard. Due to the monotonicity of maximum delay-bounded coverage, the greedy algorithm is a competitive candidate. As our first greedy algorithm, we can select the candidate sites that maximise the delay-bounded coverage gain in each iteration. When we consider to install an RSU at site *v_i_*, 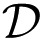
*_i_* may include some road segments that are already delay-bounded covered by the existing RSUs. Therefore, only the newly delay-bounded covered road segments are regarded as the gain of this considered RSU. We define the *g_c_*(*i*) and *g_w_*(*i*) as the delay-bounded coverage gain of installing a c-RSU and a w-RSU at site *i*, respectively. Then, we have:

(10) $$\begin{array}{c} g_{c}(i)=|D_{c}(i)\cap (E-D_{p})|\\ g_{w}(i)=|D_{w}(i)\cap (E-D_{p})| \end{array}$$

Our gain greedy algorithm for BCDP problem is presented as Algorithm 1. The major steps of Algorithm 1 are as follows. First, we compute the expect information dissemination delay *D_ij_* between any two candidate sites in Line 2, then we calculate the delay bounded coverage gain of all candidate sites for both installing c-RSUs and w-RSUs. This is accomplished in Line 4–6 and Line 7–9, respectively. After that, we find best node which has the maximal delay bounded coverage gain. This is accomplished in Line 10. Then, we determine which type of RSUs installation. In Line 11–13, we install the c-RSUs as the delay-bounded gain of the selected c-RSU is the maximum one and the total cost is not exceed the budget. If the total cost is exceed the budget, the algorithm chooses a maximal delay-bounded gain for w-RSU installation in Line 14–16. In Line 18–20, we install the w-RSUs as the delay-bounded gain of the selected w-RSU is the maximum one and the total cost is not exceed the budget. After that, we add the best node to the set of placement RSUs, and remove all the delay-bounded coverage from the set of candidate sites in Line 21–24. Finally, in line 26, we identify the placement RSUs set of our solution.



**Algorithm 1** Gain greedy algorithm
**Input:***G* = (*V*, *E*): The weighted road graph;*T*: The delay bound;*B*: The budget constraint;**Output:**

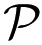
*_c_*: The placement set of installing c-RSUs;

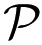
*_w_*: The placement set of installing w-RSUs;1:

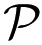
 = ∅, 

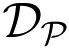
 = ∅;2:For all *i, j* ∈ *V*, initial *D_ij_* according to Equation (2);3:**while** the placement cost is not exceed the budget *B*
**and**

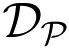
 ≠ |*E*| **do**4: **for** all unselected *v_i_* ∈ *V***do**5:  Calc c-RSUs delay-bounded coverage gain *g_c_*(*i*) according to Equation (10);6: **end for**7: **for** all unselected *v_i_* ∈ *U* and *v_i_* is with the transmission range of an existing RSU **do**8:  Calc w-RSUs delay-bounded coverage gain *g_w_*(*i*) according to Equation (10);9: **end for**10: v* = {*v_i_* ∣ max{*u_c_*(*l*), *u_w_*(*k*)}, ∀*v_i_* ∈ *V* };11: **if** install a c-RSU **and** the placement cost is not exceed the budget *B***then**12:   

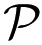
*_c_* = 

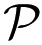
*_c_* ∪{v*};13: **else**14:  **if** install a c-RSU **and** the placement cost is exceed the budget *B***then**15:    v* = {*v_i_* ∣ max{*u_w_*(*k*)}, ∀*v_i_* ∈ *V* };16:  **end if**17: **end if**18: **if** install a w-RSU **and** the placement cost is not exceed the budget *B***then**19:  

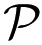
*_w_* = 

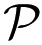
*_w_* ∪{v*};20: **end if**21: **if** install a c-RSU or w-RSU **and** the placement cost is not exceed the budget *B***then**22:  *V* = *V* \ {v*};23:   

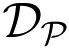
 = 

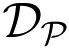
 ∪ 

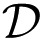
_v__*_;24: **end if**25:**end while**26:**return**

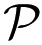
*_c_*, 

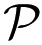
*_w_*


### 5.2. Utility Greedy Algorithm

In the gain greedy algorithm, we aim at finding the maximum delay-bounded coverage gain candidate site to install an RSU. However, the placement cost of each candidate sites is also important. This is because, although a candidate site may offer a large delay-bounded gain, it may also have a huge cost such that other candidate sites cannot be installed any c-RSU.

Consequently, our second greedy algorithm is to calculate the gain per unit cost for each iteration. We call it delay-bounded coverage utility. We denote *c_i_* and *u_i_* as the cost and delay-bounded coverage utility of installing a c-RSU in site *i*, respectively. Then, we have:

(11) $$\begin{array}{c} u_{c}(i)=\frac{\left|D_{c}(i)\cap (E-D_{p})\right|}{c_{i}}\\ u_{w}(i)=\frac{\left|D_{w}(i)\cap (E-D_{p})\right|}{c_{i}} \end{array}$$

In utility greedy algorithm, we aim at finding the maximum delay-bounded coverage utility candidate site to install an RSU. Therefore, our utility greedy algorithm for BCDP is presented as Algorithm 2.



**Algorithm 2** Utility Greedy algorithm
**Input:***G* = (*V*, *E*): The weighted road graph;*T*: The delay bound;*B*: The budget constraint;**Output:**

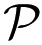
*_c_*: The placement set of installing c-RSUs;

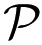
*_w_*: The placement set of installing w-RSUs;1:

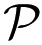
 = ∅, 

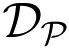
 = ∅;2:For all *i, j* ∈ *V*, initial *D_ij_* according to Equation (2);3:**while** the placement cost is not exceed the budget *B***do**4: **for** all unselected *v_i_* ∈ *V***do**5:  Calc c-RSUs delay-bounded coverage utility *u_c_*(*i*) according to Equation (11);6: **end for**7: **for** all unselected *v_i_* ∈ *U* and *v_i_* is with the transmission range of an existing RSU **do**8:  Calc w-RSUs delay-bounded coverage utility *u_w_*(*i*) according to Equation (11);9: **end for**10: v* = {*v_i_* ∣ max{*u_c_*(*l*), *u_w_*(*k*)}, ∀*v_i_* ∈ *V* };11: **if** install a c-RSU **then**12:  

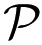
*_c_* = 

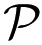
*_c_* ∪ {v*};13: **end if**14: **if** install a w-RSU **then**15:  

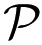
*_w_* = 

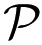
*_w_* ∪ {v*};16: **end if**17: *V* = *V* \ {v*};18: 

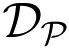
 = 

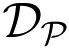
 ∪ 

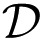
_v__*_;19:**end while**20:**return**

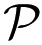
*_c_*, 

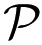
*_w_*


**Figure 3.**
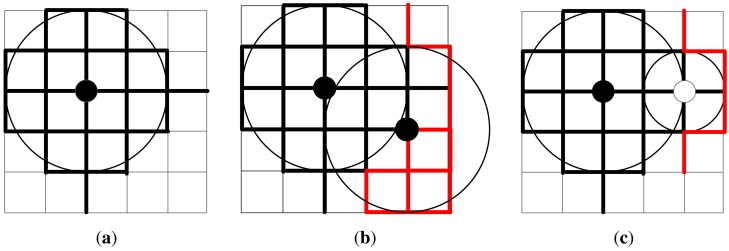
An example of the greedy algorithm. (**a**) In the first iteration, choose *v*_14_ to instal a c-RSU greedily for both Algorithms 1 and 2; (**b**) In the second iteration, choose maximal delay-bounded gain *v*_22_ to install a c-RSU greedily; (**c**) In the second iteration, choose maximal delay-bounded utility *v*_16_ to install a w-RSU greedily.

Figure 3 shows an illustrative example of the gain and utility greedy algorithms. The graph *G* has 36 candidate sites (*v*_0_ ∼ *v*_35_), which are numbered from the left to the right and from the top to the bottom. Graph *G* includes 60 road segments. Suppose the deployment cost of c-RSU and w-RSU is 2.5 and 1, respectively. The transmission range of a c-RSU and w-RSU are set to two road segments and one road segment, respectively. We assume the placement budget constrain is 5. We further assume that the expected carry-and-forward delay of each road segment is 1*min*, and the delay bound Δ is also set to 1*min*. Figure 3a shows that in the first iteration, *v*_14_ is selected to install a c-RSU, since the corresponding delay-bounded gain *g*(14) = 34 and delay-bounded coverage utility *u*(14) = 34/2.5 are both the maximum one. The 34 black thick lines are the road segments included in 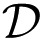
_{*v*_14_}_. So in the first iteration, we choose *v*_14_ to instal a c-RSU greedily for both Algorithms 1 and 2. Figure 3b shows in the second iteration for delay-bounded gain algorithm, *v*_22_ is chosen to install a c-RSU as the delay-bounded gain *g*(22) = 14 is the maximum one, where the red thick lines are the newly delay-bounded covered road segments. Meanwhile, the placement cost for installing this two c-RSUs is 5 and it is not exceed the budget. Figure 3c shows in the second iteration for delay-bounded utility algorithm, *v*_16_ is chosen to install a w-RSU, where the red thick lines are the newly delay-bounded covered road segments. Now the total cost is 3.5 and it is not over the budget. Note that if the budget is set to 4, the cost will be over budget for gain greedy algorithm in the second iteration. Therefore, we should not choose c-RSU to install but choose w-RSU to install. That is, we will choose *v*_16_ to install a w-RSU for gain greedy algorithm as shown in Figure 3c. In this case, the solution of gain greedy algorithm is the same as the utility greedy algorithm.

### 5.3. Analysis

Next, we analyze the approximation ratio of the Algorithm 2. We define 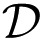
*_OPT_* as the optimal delay-bounded coverage. Let *c*(*k*) as the placement cost of the *k*-th iteration. Before we derive the approximation ratio, we have following lemma first.

#### Lemma 1

*After each iteration k, k* = 1, …, *l* + 1, *the following inequality holds*.

(12) $$\left|D_{P}^{\left(k\right)}|-|D_{P}^{\left(k-1\right)}|\geq \frac{c(k)}{B}\cdot (|D_{\text{OPT}}|-|D_{P}^{\left(k-1\right)}|\right)$$

#### Lemma 2

*After each iteration k, k* = 1, …, *l* + 1, *the following inequality holds*.

(13) $$\left|D_{P}^{\left(k\right)}|\geq \left[1-\prod_{i=1}^{k}\left(1-\frac{c(k)}{B}\right)\right]\cdot |D_{\text{OPT}}\right|$$

The detailed proof of Lemmas 1 and 2 can be found in [47].

#### Theorem 2

*The approximation ratio of the Algorithm 2 is* (1 − 1/*e*).

##### Proof

Let $C_{P}^{\left(l\right)}$ denote the total cost of our solution after *l*-th iteration. In the (*l* + 1)-th iteration, the placement cost is *c*(*l* + 1). As adding the *c*(*l* + 1) to $C_{P}^{\left(l\right)}$ exceeds the budget *B*, we have:

(14) $$C_{P}^{\left(l+1\right)}=C_{P}^{\left(l\right)}+c(l+1)>B$$

As our greedy algorithm runs (*l* + 1)-th round, according to Equation (14) and applying Lemma 2 to the (*l* + 1)-th iteration, we get:

(15) $$\begin{array}{ll} \left|D_{P}\right| & \geq \left[1-\prod_{i=1}^{k+1}\left(1-\frac{c(k+1)}{B}\right)\right]\cdot \left|D_{\text{OPT}}\right|\\ & \geq \left[1-\prod_{i=1}^{k+1}\left(1-\frac{c(k+1)}{C_{P}^{\left(l+1\right)}}\right)\right]\cdot \left|D_{\text{OPT}}\right|\\ & \geq \left[1-{\left(1-\frac{1}{1+1}\right)}^{l+1}\right]\cdot \left|D_{\text{OPT}}\right|\\ & \geq \left(1-\frac{1}{e}\right)\cdot \left|D_{\text{OPT}}\right| \end{array}$$

Then, we analyze the time complexity of the Algorithm 2 as follows.

#### Theorem 3

*The time complexity of the Algorithm 2 is O*(|*V*|^3^ + |*V*| · (|*V*| + |*E*|) · *L*), *where* |*V*| *is the number of candidate sites*, |*E*| *is the number of road segments and L is running rounds of Algorithm 2*.

##### Proof

Line 4–6 of Algorithm 2 computes the cost effectiveness for c-RSU placement. The calculation of delay-bounded coverage needs to compute *D_ij_*. The time complexity of *D_ij_* is *O*(|*V*|^3^) as we use Floyd-Warshall algorithm to compute the shortest path between any two candidate sites. The size of all unselected candidate sites is no more than |*V*|, the calculation of delay-bounded coverage will run *L* rounds and the calculation of delay-bounded coverage gain is no more than |*E*|. Therefore, The time complexity of Line 4–6 is *O*(|*V*| · |*E*| · *L*). In Line 7–9 of Algorithm 2, the time complexity is also *O*(|*V*| · |*E*| · *L*) as the number of candidate sites for w-RSUs is no more than |*V*|. Next, it iteratively chooses the site which has the maximum delay-bounded coverage utility in line 10 and the time complexity is *O*(|*V*|^2^ ·*L*). Therefore, the total time complexity is *O*(|*V*|^3^ + |*V*| · (|*V*| + |*E*|) · *L*).

## 6. Performance Evaluation

In this section, we evaluate the performance of our proposed algorithms, and present the simulation results of the algorithms in different situations.

### 6.1. Synthetic Simulation Model

In the synthetic simulation, we model the street layout of urban areas by a grid graph *G*(*V*, *E*). The candidate sites number is |*V*|, and the number of road segments is |*E*|. The length of road segment is set to 500 m, which is regarded as the length unit in the simulation. Therefore, a 10 × 10 network means the region has 10 × 10 evenly distributed candidate sites, with all sides 500 m × 10 = 5000 m long. The expected carry-and-forward delay of each road segment is set to 5 min. Since we consider the epidemic forwarding messages, we assume there is no transmission conflict and both vehicles and RSUs have infinite buffer to store packets. We define delay-bounded coverage ratio as the as the percentage of road segments that can be delay-bounded covered by the placed RSUs. We first study the delay-bounded coverage ratio, delay-bounded utility and number of RSUs under different candidate sites, different budget constraint, different cost ratio, different delay bounds and different transmission ranges ratio. We consider several algorithms as follows.

- **Random algorithm for hybrid RSUs installation (Random):** In this algorithm, we randomly choose to install either c-RSUs or w-RSUs. In each iteration, we randomly install an RSU.
- **Greedy-based algorithm for only c-RSUs installation (c-RSUs):** In this algorithm, we greedily install and only install c-RSUs.
- **Our gain greedy algorithm (Gain):** Our proposed delay-bounded gain greedy algorithm for BCDP problem, and we greedily choose the maximum gain to install either c-RSUs or w-RSUs.
- **Our utility greedy algorithm (Utility):** Our proposed delay-bounded greedy algorithm for BCDP problem, and we greedily choose the maximum utility to install either c-RSUs or w-RSUs.

### 6.2. Impact of Candidate Sites

**Figure 4.**
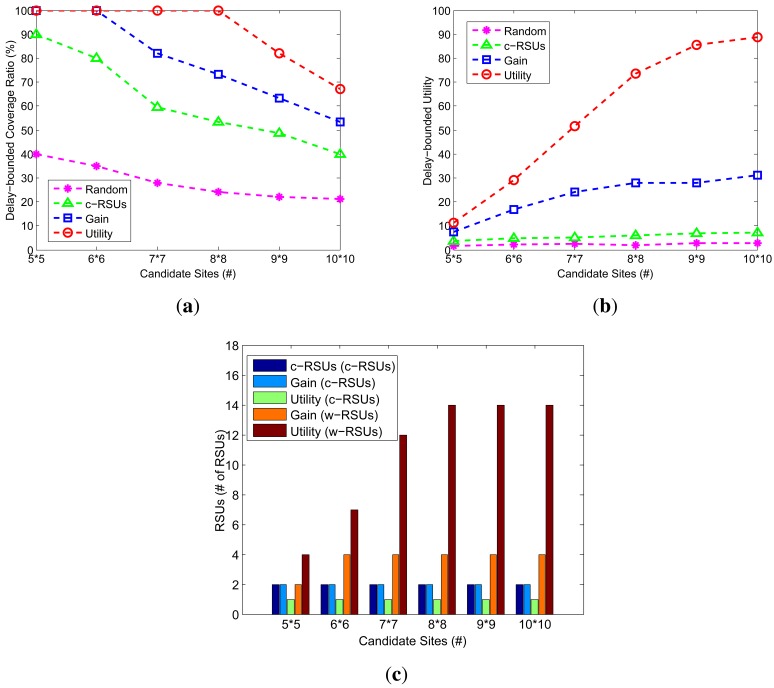
Impact of candidate sites (Budget constraint = 24, Cost ratio = 10, Transmission range of w-RSUs = 500 m, Transmission range of c-RSUs = 1 km, Delay bound = 5 min). (**a**) Coverage; (**b**) Utility; (**c**) Number of RSUs.

We investigate how the changes of candidate sites impact the performance of the different algorithms. Figure 4 shows the delay-bounded coverage, delay-bounded utility and the number of RSUs under different candidate sites of 5 × 5, 6 × 6, 7 × 7, 8 × 8, 9 × 9 and 10 × 10. The budget constraint is 24, we define the cost ratio as the ratio between c-RSUs deployment cost and w-RSUs deployment cost. The cost ratio is set to 10, the delay bound is 5 min, the transmission range of w-RSUs and c-RSUs is 500 m and 1 km, respectively. Compared with other algorithm, our two greedy algorithms always obtain the higher delay-bounded coverage and utility. Meanwhile, the larger candidate sites lead to the lower delay-bounded coverage as shown in Figure 4a,b, respectively. In Figure 4c, we plot the c-RSUs numbers of three algorithms and w-RSUs numbers of our two greedy algorithm algorithms. When the network size is small, we could only place one or two c-RSU. As the network size become larger, we could see that the larger candidate sites yield to the more w-RSUs. This is because the larger candidate sites yield to the more road segments, leading to place the larger number of RSUs and the higher deployment cost.

### 6.3. Impact of Budget Constraints

**Figure 5.**
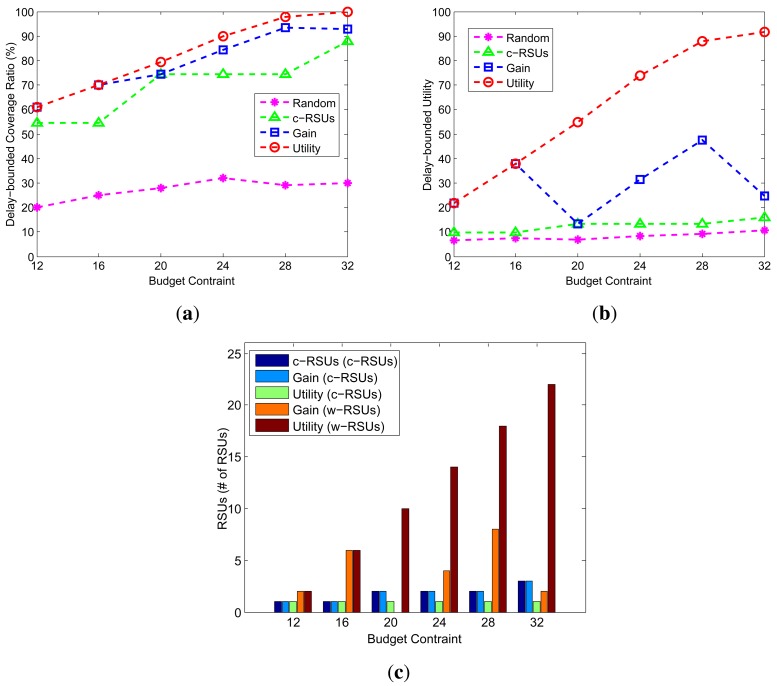
Impact of budget constraints (Candidate sites = 10 × 10, Cost ratio = 10, Transmission range of w-RSUs = 500 m, Transmission range of c-RSUs = 2 km, Delay bound = 5 min). (**a**) Coverage; (**b**) Utility; (**c**) Number of RSUs.

Figure 5 shows the delay-bounded coverage, delay-bounded utility and the number of RSUs under different budget constraints from 12 to 32 with an increment of 4. The candidate sites is 10 × 10, the cost ratio is 10, the delay bound is 5 min, the transmission range of w-RSUs and c-RSUs is 500 m and 2 km, respectively. As shown is Figure 5a,b, our two greedy algorithms always obtain the higher coverage and utility. Meanwhile, the larger budget constraint lead to the higher delay-bounded coverage. This can be understood since the larger budget constraint yield the more RSUs placement. In Figure 5c, as the budget constraint become larger, we could see that the larger c-RSUs and w-RSUs compare with the small budget constraint.

### 6.4. Impact of Cost Ratio

**Figure 6.**
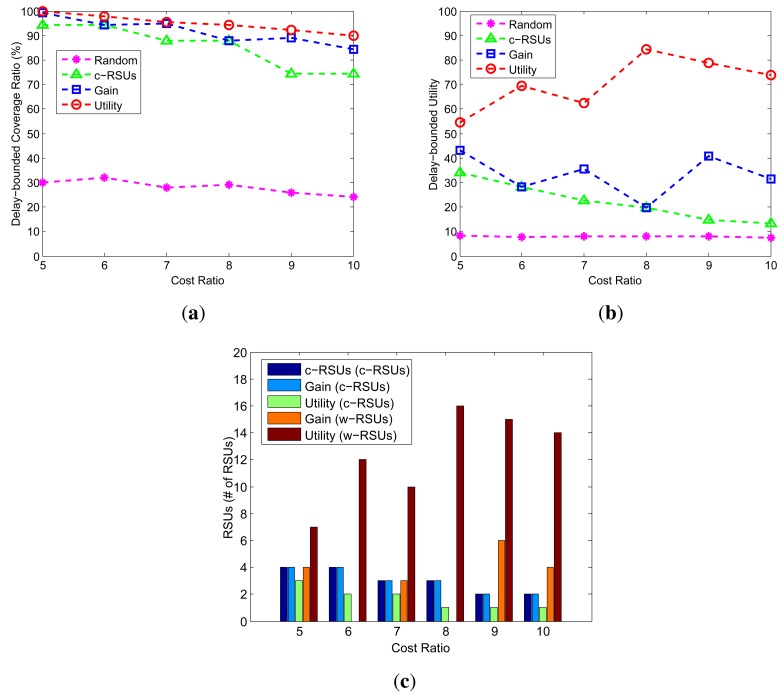
Impact of cost ratio (Candidate sites = 10 × 10, Budget constraint = 24, Transmission range of w-RSUs = 500 m, Transmission range of c-RSUs = 2 km, Delay bound = 5 min). (**a**) Coverage; (**b**) Utility; (**c**) Number of RSUs.

We investigate how the changes of cost ratio impact the performance of the different algorithms. Figure 6 shows the coverage, utility and number of RSUs under different value of cost ratio from 5 to 10 with an increment of 1. The candidate sites are 10 × 10, the budget is 24, the delay bound is 5 min, the transmission range of w-RSUs is 500 m, and the transmission of c-RSUs is 2 km. Compared with other algorithm, our two greedy algorithms always obtains the maximum delay-bounded coverage and utility. Figure 6c shows that the different algorithms calculate the number of RSUs under different cost ratio. We find that when cost ratio is small, such as 5, 6 and 7, the number of w-RSUs is smaller in Figure 6c. As the value of cost ratio increases, the number of w-RSUs decreases. This can be understood since with larger value of cost ratio, the cost of c-RSUs is larger too, and the two greedy algorithms will choose w-RSUs to install, rather than choose c-RSUs to install.

### 6.5. Impact of Delay Bounds

**Figure 7.**
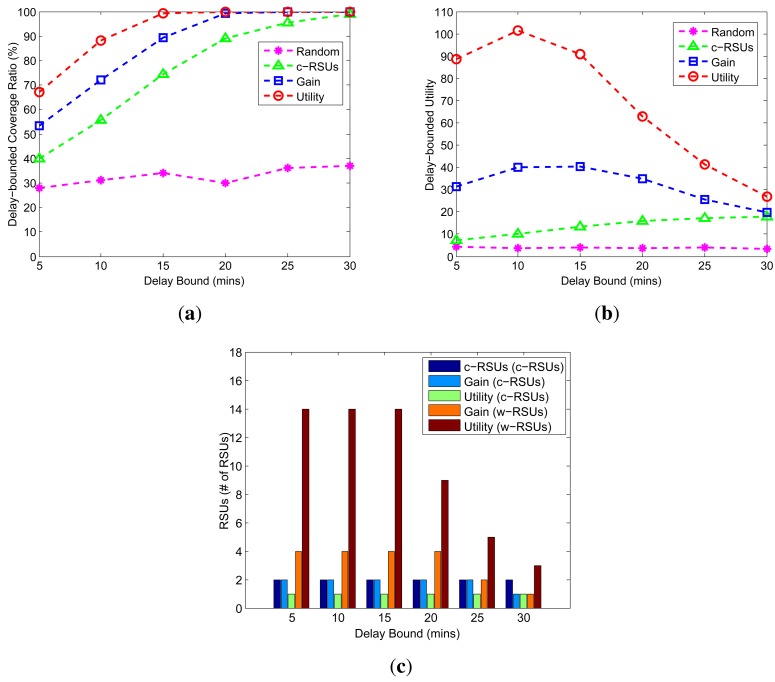
Impact of delay bounds (Candidate sites = 10 × 10, Budget constraint = 24, Transmission range of w-RSUs = 500 m, Transmission range of c-RSUs = 1 km, Cost ratio = 10). (**a**) Coverage; (**b**) Utility; (**c**) Number of RSUs.

We investigate how the changes of delay bound impact the cost, and the number of RSUs in Figure 7. The delay bounds are from 5 min to 30 min, the candidate sites are 10 × 10, the budget constraint is 24, the cost ratio is 10, the transmission range of w-RSUs is 500 m and the transmission range of c-RSUs is 1 km. As shown in the Figure 7a, for all algorithms, the longer delay bound leads to the larger delay-bounded coverage. This is because the larger delay bound increases the delay constraint, leading to the larger delay-bounded coverage. Moreover, our two greedy algorithms have almost the same performance under different value of delay bounds. As shown in Figure 7c, as the delay bound decreases, the number of c-RSUs increases smoothly, while the number of w-RSUs decreases dramatically. Therefore, with the delay bound decreases, we could choose to install more w-RSUs to meet the delay bound, rather than choose to install c-RSUs in a given area.

### 6.6. Impact of Transmission Range Ratio

**Figure 8.**
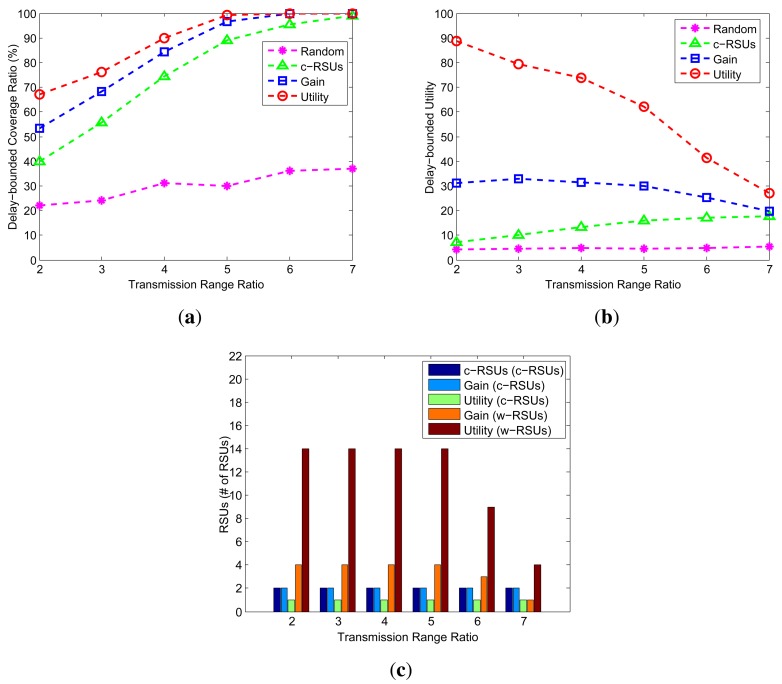
Impact of transmission range ratio (Candidate sites = 10 × 10, Budget constraint = 24, Transmission range of w-RSUs = 500 m, Cost ratio = 10, Delay bound = 5 min). (**a**) Cost; (**b**) Utility; (**c**) Number of RSUs.

The transmission range ratio is defined as the ratio between the transmission range of c-RSUs and the transmission range of w-RSUs. Figure 8 shows the different performance under different transmission range ratio. The transmission of w-RSUs is 500 m, and transmission range ratio is from 2 to 7. Therefore, the transmission of c-RSUs is 1.0 km, 1.5 km, 2 km, 2.5 km, 3 km and 3.5 km. The candidate sites are 10 × 10, the cost ratio is 10 and the delay bound is 5 min. As shown in the Figure 8a,b, even when the transmission is low, our two greedy algorithms still achieves a good performance (e.g., when the transmission range of c-RSUs is 1.0 km). As the transmission range ratio increases, the number of w-RSUs drops smoothly as shown in Figure 8c. This is because the more transmission range ratio increases, the more w-RSUs are connected with c-RSUs by wireless communication in no time and the more covered road segments we obtain, so the number of w-RSUs decreases.

### 6.7. Realistic Simulation

**Figure 9.**
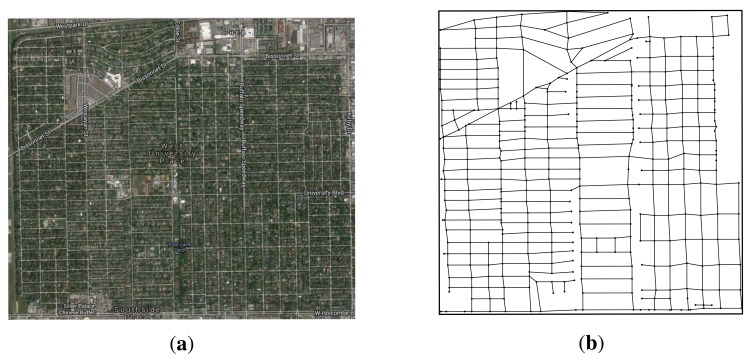
West University Place, Houston, TX, USA. (Candidate sites = 383, Road segments = 1188). (**a**) Google map; (**b**) Road network.

In the realistic simulation, we use the SUMO(Simulation of Urban MObility) [48] to convert the realistic map data to the road networks. The realistic map data is from the US Census Bureau topologically integrated geographic encoding and referencing (TIGER) database. We first obtain the downtown area of the West University Place, Houston, TX, USA from the TIGER database. Then, we use SUMO to convert this area to the road networks, and we use MOVE(MObility model generator for VEhicular networks) [49] to generate realistic mobility models for the simulations. Figure 9 shows the downtown area of West University Place, Houston, TX, USA. Figure 9a shows the satellite map of this area from Google map, and Figure 9b shows the road network in the MOVE. The network size of this area is 2.4 km × 2.4 km. There are 383 candidate sites and 1188 road segments. The expected carry-and-forward delay of each road segment is set to 5 min. The cost ratio is set to 10 unit. The delay bound is set to 5 min, the transmission range of w-RSUs and c-RSUs is 300 m and 1 km, respectively. In the simulation, we consider c-RSUs algorithm and our two algorithms. We first study the delay-bounded coverage ratio, delay-bounded utility and number of RSUs under different budget constraints. Then, we plot the placement results of this three algorithms under different budget constraints.

**Figure 10.**
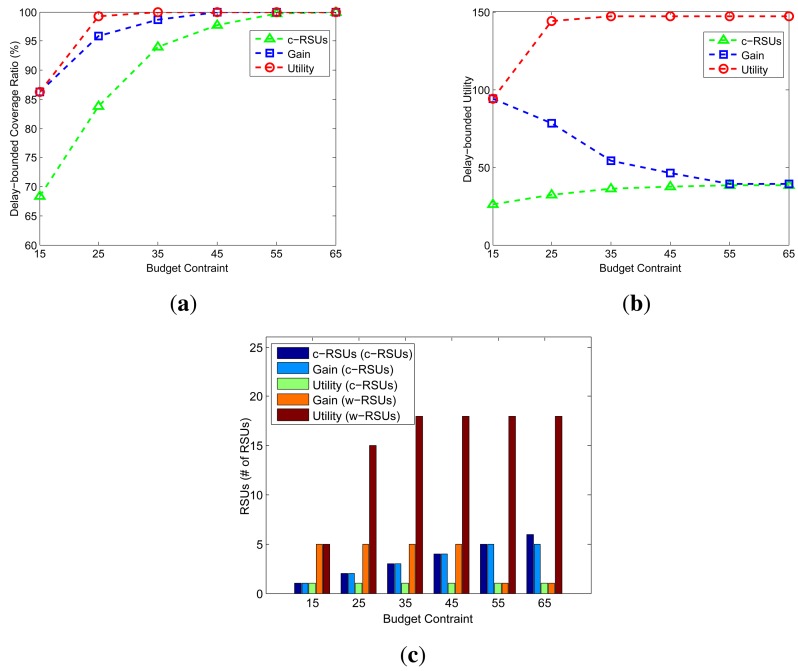
Impact of budget constraint (Candidate sites = 383, Road segments = 1188, Cost ratio = 10, Transmission range of w-RSUs = 300*m*, Transmission range of c-RSUs = 1 km, Delay bound = 5 min). **(a)** Coverage; **(b)** Utility; **(c)** Number of RSUs.

Figure 10 shows the delay-bounded coverage, delay-bounded utility and the number of RSUs under different budget constraints from 15 to 65 with an increment of 10. As shown is Figure 10a,b, our two greedy algorithms always obtain the higher coverage and utility. When the budget constraint is above 35 and 45, our utility and gain greedy algorithms could cover the whole area within the given delay bound. Notice that as the budget constraint become larger, the utility of our gain greedy algorithm drops smoothly. This is because the more budget constraint, the more c-RSUs are installed, so the delay-bounded utility decreases. We could see the number of c-RSUs and w-RSUs in Figure 10c. The budget is 15, our gain greedy algorithm place one c-RSU and five w-RSUs. When the budget constraint up to 25, our gain greedy algorithm could place two c-RSU and five w-RSUs.

### 6.8. RSUs Placement Results

**Figure 11.**
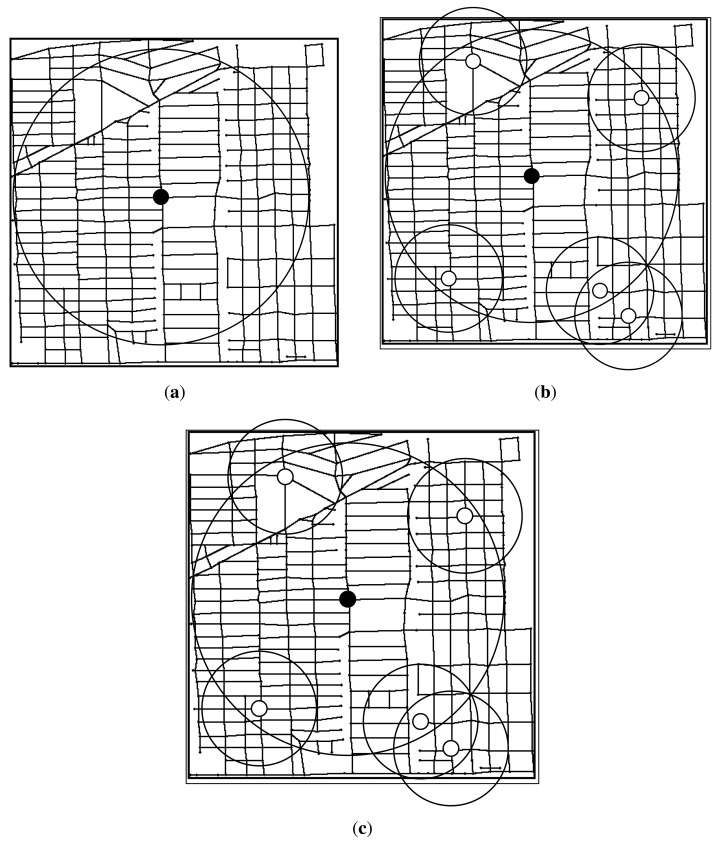
RSUs placement results under budget constraint = 15 (Candidate sites = 383, Road segments = 1188, Transmission range of w-RSUs = 300*m*, Transmission range of c-RSUs = 1 km, Cost ratio = 10, Delay bound = 5 min). (**a**) c-RSUs algorithm; (**b**) Gain greedy algorithm; (**c**) Utility greedy algorithm.

Next, we plot the RSUs placement result under different budget constraints. We first plot the RSUs placement result of three algorithms when budget constraint is set to 15. As shown in Figure 11, the transmission range of w-RSUs is 300 *m*, the transmission range of c-RSUs is 1 km, the cost ratio is 10 and the delay bound is 5 min. As placement cost of installing a c-RSU is 10 unit, we could only install one c-RSU for the only installing c-RSUs algorithm. So we install a c-RSU with maximum gain as shown in Figure 11a. As shown in Figure 11b,c, our gain greedy algorithm and utility greedy algorithm have the same placement strategy. Our two algorithms all place one c-RSU and five w-RSUs when budget constraint is 15 unit. We could also obtain this result in Figure 10, the delay-bounded coverage ratio, the delay-bounded utility and the number of RSUs of our two algorithms are the same when the budget constraint is 15.

**Figure 12.**
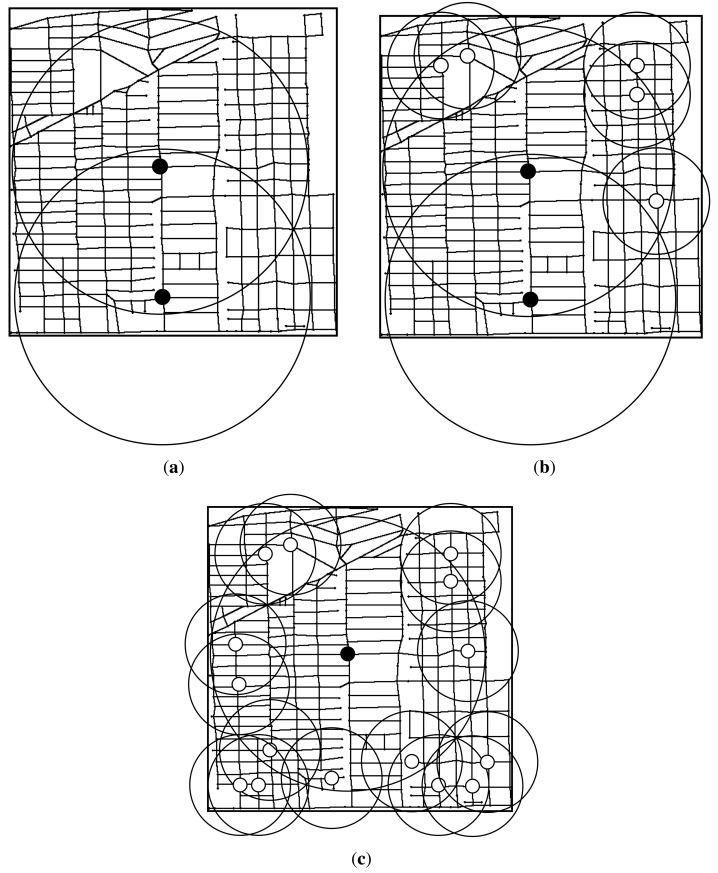
RSUs placement results under budget constraint = 25 (Candidate sites = 383, Road segments = 1188, Transmission range of w-RSUs = 300 m, Transmission range of c-RSUs = 1 km, Cost ratio = 10, Delay bound = 5 min). (**a**) c-RSUs algorithm; (**b**) Gain greedy algorithm; (**c**) Utility greedy algorithm.

Then, we plot the RSUs placement result when budget constraints is set to 25 unit in Figure 12. As shown in Figure 12a, we could only install two c-RSU for the only installing c-RSUs algorithm, and the delay-bounded ratio increases to 83.8%. For our gain greedy algorithm, we always install the maximum delay-bounded coverage gain to install. In the early stages of our gain greedy algorithm, we always install the c-RSUs as the c-RSUs cover more road segments. So we place two c-RSUs as shown in Figure 12b. Then, we choose the candidates to place w-RSUs, and we place five w-RSUs at the end of the gain greedy algorithm. The delay-bounded coverage ratio increases to 95.8%. For our utility greedy algorithm, we choose the candidate with the maximum delay-bounded coverage utility to place an RSU. As shown in Figure 12c, we place one c-RSU and fifteen w-RSUs at the end of the utility greedy algorithm. The delay-bounded coverage ratio increases to 99.2%. We could see that the delay-bounded coverage area of our utility algorithm is larger than our gain algorithm and c-RSUs algorithm.

**Figure 13.**
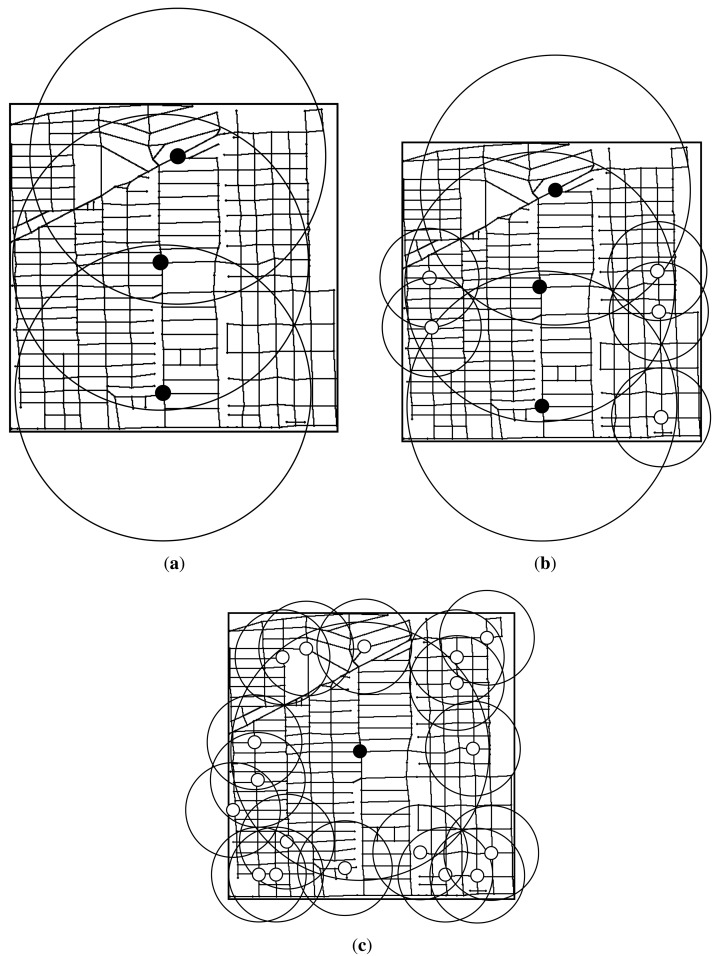
RSUs placement results under budget constraint = 35 (Candidate sites = 383, Road segments = 1188, Transmission range of w-RSUs = 300*m*, Transmission range of c-RSUs = 1 km, Cost ratio = 10, Delay bound = 5 min). (**a**) c-RSUs algorithm; (**b**) Gain greedy algorithm; (**c**) Utility greedy algorithm.

In Figure 13, we plot the RSUs placement result when budget constraint is set to 35 unit. As shown in Figure 13a, we install three c-RSU for the only installing c-RSUs algorithm, and the delay-bounded coverage ratio is 94.0%. For our gain greedy algorithm, we place three c-RSUs as shown in Figure 13b. In this case, we can't continue place c-RSUs as the total placement cost will be over the budget. Then, we choose the candidates to place w-RSUs, and we place five w-RSUs at the end of the gain greedy algorithm. The delay-bounded coverage ratio increases to 98.7%. For our utility greedy algorithm in Figure 13c, we continue place another three w-RSUs and the coverage ratio increases to 100%. That means all the road segments could be covered by the installing RSUs within the given delay bound. We call this *full coverage*. Then, we do not need to place any more RSUs. Therefore, we place one c-RSU and eighteen w-RSUs at the end of the utility greedy algorithm.

**Figure 14.**
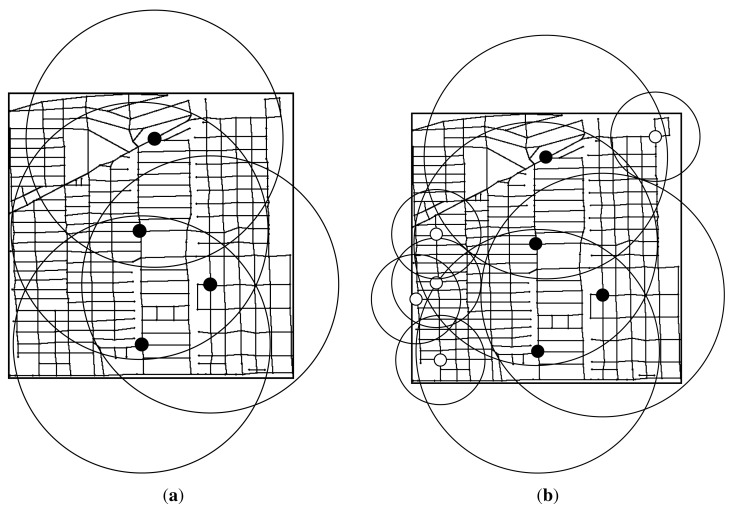
RSUs placement results under budget constraint = 45 (Candidate sites = 383, Road segments = 1188, Transmission range of w-RSUs = 300*m*, Transmission range of c-RSUs = 1 km, Cost ratio = 10, Delay bound = 5 min). (**a**) c-RSUs algorithm; (**b**) Gain greedy algorithm.

In Figure 14, we plot the RSUs placement result when budget constraint is set to 45 unit. As our utility greedy algorithm do not place any more RSUs, the RSUs placement result is the same as the Figure 13c shown. We only plot the the RSUs placement result of c-RSUs algorithm and gain greedy algorithm. As shown in Figure 14a, we install four c-RSU for the only installing c-RSUs algorithm, and the delay-bounded coverage ratio increase to 97.7%. For our gain greedy algorithm, we place four c-RSUs and five w-RSUs as shown in Figure 14b. The delay-bounded coverage ratio also increases to 100%. That means the RSUs placement result with *full coverage* for our the gain greedy algorithm when budget is over 45.

**Figure 15.**
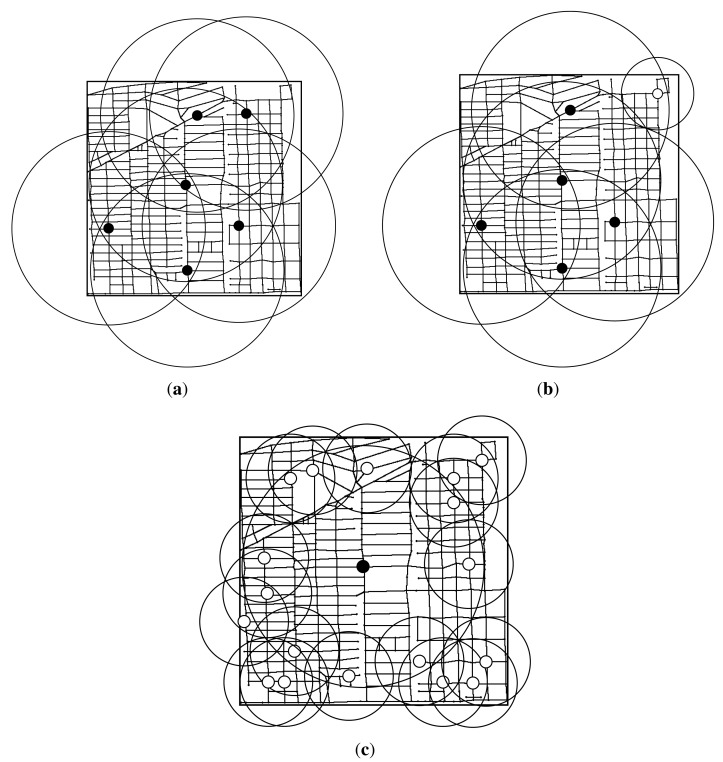
RSUs placement results of full coverage (Candidate sites = 383, Road segments = 1188, Transmission range of w-RSUs = 300 *m*, Transmission range of c-RSUs = 1 km, Cost ratio = 10, Delay bound = 5 min). (**a**) c-RSUs algorithm for full coverage, budget ≥ 60; (**b**) Gain greedy algorithm for full coverage, budget ≥ 51; (**c**) Utility greedy algorithm for full coverage, budget ≥ 28.

Finally, we plot the RSUs placement result with *full coverage* in Figure 15. As shown in Figure 15a, we should install six c-RSUs for the c-RSUs algorithm to achieve the *full coverage*. Obvious, the c-RSUs algorithm to cover boundary road segments is not very cost-effective. For our gain greedy algorithm, we have two placement strategy. As shown in Figure 14b, when budget is above 45 and below 50, we place four c-RSUs and five w-RSUs and we obtain the *full coverage*. While budget is 51 or above 51, we place five c-RSUs and one w-RSU and we also obtain the *full coverage* as shown in Figure 15b. The placement cost of this two placement strategy is 45 and 51, respectively. As shown in Figure 15c, we only place one c-RSU and eighteen w-RSUs to obtain the full coverage. The placement cost is only 28. Obviously, the utility greedy strategy is superior to other placement strategies.

## 7. Conclusions

In this paper, we investigate the budget constrained and delay-bounded RSUs placement problem in vehicular *ad hoc* networks, which finds maximum delay-bounded coverage and meanwhile a message sent from the c-RSUs can be disseminated to more vehicles in a given delay bound. First, we propose a delay-bounded coverage model for RSUs placement, which inherently provides a good balance between delay and coverage. Next, we formulate BCDP problem to the budgeted maximum coverage problem and prove that this problem is NP-hard. Finally, we propose two heuristic algorithms to solve the BCDP problem. Simulation results show effectiveness of our methods under different environments. As a future work, we will explore how to effectively deploy RSUs in VANET under different forwarding models.
